# Prevalence and correlates of sexual intercourse among sexually active in-school adolescents: an analysis of five sub-Sahara African countries for the adolescent’s sexual health policy implications

**DOI:** 10.1186/s12889-019-7632-1

**Published:** 2019-10-12

**Authors:** Festo K. Shayo, Mariam H. Kalomo

**Affiliations:** 1 Department of Internal Medicine, Muhimbili University of Health and Allied Sciences, P.O Box 65001, Dar es Salaam, Tanzania; 2 Division of Public Health, Department of Global Health Entrepreneurship, Graduate School of Tokyo Medical and Dental University, 1-5-45 Yushima, Bunkyo-ku, Tokyo, 113-8510 Japan; 3 Department of Non Communicable Disease, Ministry of Health, Community Development, Gender, Elderly, and Children (MoHCDGEC), Dodoma, Tanzania

**Keywords:** Adolescents, Sexual intercourse, Substance use, Parental connectedness, HIV/AIDS, Sub-Saharan Africa

## Abstract

**Background:**

Early and unprotected sex with multiple partners among adolescents carries a high risk of acquiring HIV infections, other sexually transmitted infections as well as high rates of teenage pregnancy. Sub-Saharan Africa (SSA) has a higher burden of HIV/AIDS: the leading cause of deaths among adolescents. We estimated the prevalence and examined the correlates of sexual intercourse among in-school adolescents of SSA. The purpose is to inform the public health programs dedicated to tackling the burden of HIV/AIDS.

**Methods:**

We did a secondary analysis of the Global School-based Student Health Surveys (GSHS) datasets pooled from five SSA countries Benin, Mozambique, Namibia, Seychelles, and Tanzania. Our current analysis included a sample of 15,318 in-school adolescents. The primary independent variables were ever had sexual intercourse and sex with multiple partners, while the dependent variables were smoking cigarettes, alcohol use, use of marijuana and amphetamine, and parental connectedness. We performed descriptive statistics, and multivariate logistic regression stratified by gender using SPSS Complex Sample Statistics. A *p*-value of less than 0.05 was considered statistically significant at 95% confidence intervals.

**Results:**

Out of 15,318 participants, the overall prevalence of ever had sexual intercourse and sex with multiple partners were 43·5% (6670) and 20·9% (3204), respectively. In overall and across each country, male adolescents had a significantly higher proportion of sex with multiple partners than female adolescents, *p* < 0·001. The predictors of sexual intercourse with multiple partners in both male and females were smoking cigarettes, alcohol use, and use of marijuana and amphetamine. Female adolescents who smoked cigarettes and used marijuana had a significant likelihood of sex with multiple partners than male adolescents: [aOR 3.6, 95% CI: 2.6-5.1] vs [aOR 2.1, 95% CI: 1.7-2.7] and [aOR 2.4, 95% CI: 1.6-3.7] vs [aOR 1.9, 95% CI: 1·3-2·7], respectively.

**Conclusions:**

Adolescents sexual intercourse and more especially sex with multiple partners was prevalent and strongly correlated with substance use. However, the correlation was higher among female adolescents than male adolescents. A customized public health intervention that targets multiple risk factors concurrently may benefit adolescents with clustering of sexual and non-sexual risk-taking behaviors.

## Background

Adolescence period has different critical transitions such as physical, economic, and psychosocial development. Moreover, it is a preferred period for initiating sexual and reproductive health promotion programs [1]. Adolescence sexuality is among the risk-taking behaviors in this age group [2]. The immaturity of the prefrontal cortex in adolescents is associated with heightened impulsivity and inability to have a wise plan or forecast the consequences of risk-taking behaviors [3] such as sexual intercourse. Early age at sexual intercourse is a strong predictor of future poor sexual health, including other health-related risk behaviors among adolescents [3–5]. Sexual intercourse in adolescence increases the risk of acquiring HIV infections, sexually transmitted infections (STIs) [5–7], and unintended teenage pregnancy [7–9]. Moreover, it is associated with unprotected sexual intercourse [10], having multiple sexual partners and other health-related risks behaviors, later in life [5, 8, 11]. In SSA, up to 25% of adolescents aged 15-19 years have ever had sexual intercourse before the age of 15 years, although this can vary across the countries [12].

Globally, the number of people living with HIV/AIDS is declining across all age groups: for instance, from 3.4 million in 1996 to 1.8 million in 2017 there was a decline from to, respectively [13]. However, SSA is the most affected region by HIV/AIDS accounting for over two-thirds of the daily global total new HIV infections [10]. There is evidence that 45% of the global new HIV infections occur in SSA, and 53% of people living with HIV are from this region [13]. Adolescents aged 15-24 years have a higher rate of HIV infections and more than 50% of new infections [11, 12]. About 70% of all global AIDS-related mortality occur in SSA [12] despite a decline in HIV incidence and mortality by 30 and 45% in the year 2010 and 2017, respectively [13]. In SSA, HIV/AIDS is the leading cause of deaths among adolescents aged 10-19 years [14].

Young women and adolescent girls in SSA have a disproportionally high risk of HIV infections because of the existing gender-based violence, inequalities, and physiological factors [13]. Nevertheless, some of the countries in SSA have laws and policies which discourage the adolescent girls from acquiring parental consent in accessing sexual and reproductive health [13]. Moreover, the laws that govern the adolescents HIV testing varies across the countries of SSA [15]. World Health Organization (WHO), recommends HIV testing, counseling, and access to HIV services for all adolescents in high burden setting [16]. The recent studies in SSA, have reported the low prevalence of HIV testing among adolescents aged 15-19 years: 23 and 16% among girls and boys, respectively [17, 18]. In those studies, the following were among the challenges to HIV testing and counseling: legal and policy restricted age-of-consent to HIV testing and counseling, difficult in accessing testing and counseling services, and fear of social stigma [17].

The findings from 18 HIV prevention studies showed that STIs is prevalent in SSA: higher among younger women (15-24-year-old) than older women (25-49-year-old). The average prevalence of gonorrhea for women aged 15-24 years, was found to range from 4.6% in South Africa to 8.2% in East Africa [19]. Moreover, the prevalence of at least one STI among HIV-infected adolescents aged 13–19 years was found to be 28% in Kenya [20] and 13.5% in Ethiopia [21]: females were more affected than male adolescents.

Adolescent pregnancy has significant adverse maternal and perinatal outcomes. The incidence of adolescent pregnancy is on the rise in low and middle-income countries (LMICs), including SSA [22]. In SSA, the proportion of adolescent pregnancy is highest with over 200 births per 1000 girls age 15–19 years [23]. The pooled prevalence of adolescent pregnancy from 52 studies was reported to be 18.8% for Africa and 19.3% for the SSA region. Poor parental-child communication on sexual and reproductive health was among the reported risk factor for adolescent pregnancy [24].

It is not uncommon to find a clustering of risk-taking behaviors such as substance use: alcohol drinking, use of tobacco, marijuana, and amphetamines among adolescents [25]. In SSA, the co-occurrence of risk-taking behaviors such as the use of alcohol, tobacco, and illicit drugs are reported to drive more sexual intercourse behaviors among adolescents [2, 22, 26]. For instance, a study conducted in four countries of SSA revealed that adolescents who drink alcohol tend to have more than one sexual partner compared to their counterparts [27]. A school-based health survey in Kenya found that adolescents who engaged in sexual intercourse were more likely to use illicit drugs, drink alcohol, and smoke cigarettes [28]. Although risk-taking behaviors among adolescents may occur in clusters, they are easily forgotten by the programs that target only a single domain, for instance, reproductive health alone [2]. The single-domain approach may not be effective in tackling the global adolescent’s health, given the clustering of sexual and non-sexual risk-taking behaviors. Therefore, multiple-domain prevention programs: an intervention that targets multiple risk behaviors concurrently may be needed. In SSA, there is limited information about the correlates between the clustering of non-sexual risk-taking behaviors and sexual intercourse behaviors among adolescents. Therefore, our current study sought to estimate the prevalence and correlates of sexual intercourse among in-school adolescents from five SSA countries. The purpose is to inform the public health programs dedicated to fighting HIV/AIDS.

## Method

### Data source

We did a secondary analysis of the Global School-based Student Health Surveys (GSHS) datasets pooled from five SSA countries Benin, Mozambique, Namibia, Seychelles, and Tanzania. Our current analysis included a sample of 15,318 in-school adolescents. The primary independent variables were ever had sexual intercourse and sex with multiple partners, while the dependent variables were smoking cigarettes, alcohol use, use of marijuana and amphetamine, and parental connectedness. We performed descriptive statistics, and multivariate logistic regression stratified by gender using SPSS Complex Sample Statistics. A *p*-value of less than 0.05 was considered statistically significant at 95% confidence intervals.

We did a secondary analysis of the Global School-based Student Health Surveys (GSHS) datasets pooled from five SSA countries Benin, Mozambique, Namibia, Seychelles, and Tanzania. We purposely selected those countries from among other SSA countries because they did their surveys near the same year period 2013-2016. Moreover, each country included the question about sexual behaviors, substance use behaviors (cigarette smoking, alcohol drinking, use of marijuana and amphetamine) and parental connectedness in the survey. More details about the GSHS dataset files can be accessed through https:www.who.int/ncds/surveillance/gshs/datasets/en/.

The GSHS is a self-administered, school-based survey developed by the WHO in collaboration with the United Nations Children Fund (UNICEF), the Joint United Nations Programme on HIV/AIDS (UNAIDS), and the United Nations Educational, Scientific and Cultural Organization (UNESCO). The financial and technical assistance was provided by the US Centre for Disease Control and Prevention (CDC). The purpose of GSHS is to collect data about risk-taking behaviors including sexual behaviors that contribute to HIV infection, other sexually transmitted infections, and unintended pregnancy from students aged 13 to 17 years [28].

### Sampling technique, data collection, and sample size

The GSHS surveys used a two-stage cluster sampling to obtain a representative sample of students in each country. The first stage primary sampling units, schools were selected with a probability proportional to their enrolment size. The second stage involved a sampling of a systematic sample of classes in the selected schools. All students in the selected classes were eligible and invited to participate. Moreover, they were clearly explained that they are free not to participate or respond to any question in the questionnaire. Survey questions were translated into an appropriate language of instructions for the students and were pilot tested for comprehension. Students completed a self-administered questionnaire during one classroom period without personal identifiers. The current analysis used pooled data from Benin, Mozambique, Namibia, Seychelles, and Tanzania surveys administered during 2016, 2015, 2013, 2015, and 2014, respectively. A total of 15,318 students from the five countries completed the survey. For more details, see Additional file 1: Table S1.

### Measures of variables

#### Dependent variables

The sexual intercourse variables were: (i) “Percentage of students who ever had sexual intercourse” The variable was dichotomized as Yes and No. (ii)“ Percentage of students who had sexual intercourse with two or more persons (during their life)” The variable was dichotomized as Yes and No.

#### Independent variables

Substance use variables: (i) Currently smoked cigarettes: “During the past 30 days, on how many days did you smoke cigarettes? The variable was dichotomized as “0 days“ and “1 ≥ day“. (ii) Currently drank alcohol: “During the past 30 days, on how many days did you have at least one drink containing alcohol? The variable was dichotomized as “0 days” and “1 ≥ day”. (iii) Currently used Marijuana (cannabis): “During the past 30 days, how many times have you used marijuana? The variable was dichotomized as “0 days” and “1 ≥ day“. (iv) use of amphetamine/methamphetamine: “During your life, how many times have you used amphetamines or methamphetamines (also known as stimulants not prescribed by a doctor)?“ The variable was dichotomized as “0 days” and “1 ≥ day”.

Parental connectedness: For the purpose of the current study it is defined as how often the parents or guardians understand the problems or worries of their children. The students were asked: “During the past 30 days, how often did your parents or guardians understand your problems or worries?” The variable was dichotomized as “sometimes to always” and “never or rarely”.

We also included sociodemographic variable; gender dichotomized as male and female, age, and class grades (grade 6 to 12).

### Data process and analysis

We considered the appropriateness of combining the GSHS datasets across the five countries, including sampling design and error and non-sampling error. Previous studies have shown that combining country-based survey data to regional estimate is realistic [29]. The sampling design was similar across the selected countries, and participants within each country were done at two levels: school and class. Non-sampling errors were examined using survey overall response rates, ranged from 78% (Benin) to 89% (Namibia): see Additional file 1: Table S1. Following the above consideration, we pooled the data from the five countries GSHS datasets.

The GSHS dataset has missing data as a result of nonresponses. We observed that the data were missing at random pattern. Therefore, we used multiple imputations [30–32] to examine a possibility of bias between findings of the imputed dataset and that of the original dataset. We found that there were no differences in outcomes of interest when we replaced the missing data with imputed data. Therefore, a significant measure of association in both the bivariate and multivariate analyses was maintained despite imputation. We run a maximum of five imputations to allow for > 97% efficiency. Different studies elsewhere have applied multiple imputations technique to account for missing data [33–37].

Our current analysis used a Complex Samples Statistics command of SPSS version 22 because of the complex sampling design nature of the GSHS. We used a chi-square χ^2^ to compare the differences between the categorical variables, and a multivariate logistic regression stratified by gender to explain the association between independent and dependent variables. Each of the variables in the model was adjusted for the rest of all other variables. The category age was analyzed as a continuous variable. A *p*-value of less than 0.05 was considered statistically significant at 95% confidence intervals.

## Results

Table 1 represents the prevalence of sexual and non-sexual risk-taking behaviors among in-school adolescents stratified by gender.

**Table 1:** Prevalence of sexual and non-sexual risk-taking behaviors among in-school adolescents stratified by gender

Country	Variables		Total *n* (%)	Gender		*P*-value
				Male *n* (%)	Female *n* (%)	
Benin (*n* = 2536)	Ever had sexual intercourse	Yes	1215 (47.9)	799 (31.5)	416 (16.4)	< 0.001
	Sex with multiple partners	Yes	650 (25·6)	541 (21.3)	109 (4.3)	< 0.001
	Currently smoked cigarettes	Yes	109 (4.3)	87 (3.4)	22 (0.9)	< 0.001
	Currently drank alcohol	Yes	1015 (40.0)	593 (23.4)	422 (16.6)	0.003
	Currently used marijuana	Yes	24 (0.9)	20 (0.8)	4 (0.1)	0.005
	Ever used amphetamine	Yes	49 (1.9)	37 (1.5)	12 (0.4)	0.002
	Parental connectedness	Yes	835 (32.9)	438 (17.3)	397 (15.6)	0.23
Mozambique (*n* = 1918)	Ever had sexual intercourse	Yes	1149 (59·9)	706 (36.8)	443 (23.1)	< 0.001
	Sex with multiple partners	Yes	544 (28·4)	411 (21.4)	133 (7.0)	< 0.001
	Currently smoked cigarettes	Yes	55 (2.8)	34 (1.7)	21 (1.1)	0.46
	Currently drank alcohol	Yes	263 (13.7)	145 (7.6)	118 (6.1)	0.57
	Currently used marijuana	Yes	30 (1.6)	23 (1.2)	7 (0.4)	0.02
	Ever used amphetamine	Yes	28 (1.5)	17 (0.9)	11 (0.6)	0.34
	Parental connectedness	Yes	854 (44.5)	450 (23.5)	404 (21.0)	0.74
Namibia (*n* = 4531)	Ever had sexual intercourse	Yes	2416 (53.3)	1389 (30.7)	1027 (22.6)	< 0.001
	Sex with multiple partners	Yes	1174 (25.9)	810 (17.9)	364 (8.0)	< 0.001
	Currently smoked cigarettes	Yes	443 (9.8)	300 (6.6)	143 (3.2)	< 0.001
	Currently drank alcohol	Yes	1476 (32.6)	823 (18.2)	653 (14.4)	< 0.001
	Currently used marijuana	Yes	243 (5.4)	152 (3.3)	91 (2.1)	< 0.001
	Ever used amphetamine	Yes	256 (5.6)	159 (3.5)	97 (2.1)	< 0.001
	Parental connectedness	Yes	1852 (40.9)	841 (18.6)	1011 (22.3)	0.04
Seychelles (*n* = 2540)	Ever had sexual intercourse	Yes	1048 (41.3)	555 (21.9)	493 (19.4)	< 0.001
	Sex with multiple partners	Yes	584 (23.0)	322 (12.7)	262 (10.3)	0.001
	Currently smoked cigarettes	Yes	507 (20.0)	302 (11.9)	205 (8.1)	< 0.001
	Currently drank alcohol	Yes	1177 (46.3)	549 (21.6)	628 (24.7)	0.55
	Currently used marijuana	Yes	249 (9.8)	171 (6.7)	78 (3.1)	< 0.001
	Ever used amphetamine	Yes	128 (5.0)	89 (3.5)	39 (1.5)	< 0.001
	Parental connectedness	Yes	810 (31.9)	376 (14.8)	434 (17.1)	0.55
Tanzania (*n* = 3793)	Ever had sexual intercourse	Yes	842 (22.2)	529 (13.9)	313 (8.3)	< 0.001
	Sex with multiple partners	Yes	252 (6·6)	183 (4.8)	69 (1.8)	< 0.001
	Currently smoked cigarettes	Yes	177 (4.7)	99 (2.6)	78 (2.1)	0.04
	Currently drank alcohol	Yes	190 (5.0)	101 (2.7)	89 (2.3)	0.16
	Currently used marijuana	Yes	100 (2.6)	51 (1.3)	49 (1.3)	0.99
	Ever used amphetamine	Yes	118 (3.1)	55 (1.4)	63 (1.7)	0.56
	Parental connectedness	Yes	1456 (38.4)	683 (18.0)	773 (20.4)	0.26
Overall (*N* = 15,318)	Ever had sexual intercourse	Yes	6670 (43·5)	3978 (26.0)	2692 (17.5)	< 0.001
	Sex with multiple partners	Yes	3204 (20·9)	2267 (14.8)	937 (6.1)	< 0.001
	Currently smoked cigarettes	Yes	1291 (8.4)	822 (5.3)	469 (3.1)	< 0.001
	Currently drank alcohol	Yes	4121 (26.9)	2211 (14.4)	1910 (12.5)	< 0.001
	Currently used marijuana	Yes	646 (4.2)	417 (2.7)	229 (1.5)	< 0.001
	Ever used amphetamine	Yes	579 (3.7)	357 (2.3)	222 (1.4)	< 0.001
	Parental connectedness	Yes	5807 (37.9)	2788 (18.2)	3019 (19.7)	0.01

Note: Sex with multiple partners = Sexual intercourse with ≥2 persons

Of the 15,318 participants, the overall prevalence of ever had sexual intercourse and sex with multiple partners were 43·5% (6670) and 20·9% (3204), respectively. In overall and across each country, male adolescents had a significantly higher proportion of sex with multiple partners than female adolescents.

Table 2 represents multivariate logistic regression for predictors of sexual intercourse and sex with multiple partners among in-school adolescents.

**Table 2:** Multivariate logistic regression for predictors of sexual intercourse and sex with multiple partners among in-school adolescents stratified by gender (*N* = 15,318)

Variable		Ever had sexual intercourse		Sex with ≥ 2 persons	
	Gender	OR [95% CI]	aOR [95% CI]	OR [95% CI]	aOR [95% CI]
^a^Current smoked cigarette (No)					
Yes	Male	3.2 [1·7-6.2]**	1·9 [1.3-2.8]**	3.2 [2.7-3.9]***	2.1 [1.7-2.7]***
	Female	4.4 [2·7-7.3]***	3.0 [2.2-4.2]***	6.3 [4.7-8.6***	3.6 [2.6-5.1]***
^a^Currently drank alcohol (No)					
Yes	Male	3.0 [2.4-3.8]***	2·1 [1.8-2.4]***	3·2 [2·7-3.9]***	2·3 [1·9-2·7]***
	Female	2.5 [2.1-2.9]***	1.7 [1.5-2.0]***	3·4 [2·5-4.8]***	2·2 [1·7-2·9]***
^a^Currently used marijuana (No)					
Yes	Male	5.8 [1.3-26.5]*	2.2 [0.9-5.7]	3.6 [2.8-4.8]***	1.9 [1·3-2·7]**
	Female	6.2 [1.9-19.9]*	1.9 [0.8-4.5]	7.7 [5.8-10.1]***	2.4 [1.6-3.7]***
^a^Ever used amphetamine (No)					
Yes	Male	6·8 [1·0-45.2]*	3.8 [0.8-19.0]	2.8 [2.2-3.6]***	1·3 [1·0-1.8]
	Female	7.2 [1.9-26.7]*	4.1 [1.4-12.6]*	5.6 [4.2-7.6]***	1·8 [1·1-2.9]*
^a^Parental connectedness (No)					
Yes	Male	0·8 [0·7-0·9]**	0·9 [0·8-1.0]	0·9 [0·8-1.0]	1.0 [0.9-1.1]
	Female	0·7 [0·6-0·8]***	0·7 [0·7-0·8]***	0·6 [0·5-0·8]***	0.7 [0.6-0.9]**
Age	Male	1·3 [1·3-1·4]***	1.4 [1.3-1.4]***	1·4 [1·3-1·5]***	1·4 [1·3-1·5]***
	Female	1·4 [1·3-1·4]***	1.4 [1.4-1.5]***	1·3 [1·2-1·4]***	1·4 [1·2-1·5]***

Note: *OR* odds ratio, *aOR* adjusted odds ratio

*P < 0·05, **P < 0·01, ***P < 0·001

^a^Adjusted for all variables in the model, sex with ≥2 persons = sexual intercourse with multiple persons

For both genders, the predictors of sexual intercourse with multiple partners were currently smoked cigarette, currently drank alcohol, and currently used marijuana. Female adolescents who currently smoked cigarettes and used marijuana had a significant likelihood of sex with multiple partners than male adolescents: [aOR 3.6, 95% CI: 2.6-5.1] vs [aOR 2.1, 95% CI: 1.7-2.7] and [aOR 2.4, 95% CI: 1.6-3.7] vs [aOR 1.9, 95% CI: 1·3-2·7], respectively. Only female adolescents with parental connectedness were significantly less likely to have sex with multiple partners.

## Discussion

In our current study, we estimated the prevalence of sexual intercourse and sex with multiple partners and examined their correlates with substance use among in-school adolescents. We used a representative sample size of the GSHS pooled from the five SSA countries.

Our study found that about half of the adolescents reported having engaged in sexual intercourse. More importantly, we found that about one-fourth of adolescents have ever involved in sexual intercourse with multiple partners. Previous studies in SSA have found a comparable prevalence of sex with multiple partners among adolescents, but with variation across the countries [38]. Unprotected and early age at sexual intercourse and more especially sex with multiple partners carries a high risk of acquiring HIV infections, other STIs, and teenage pregnancy [5–9]. Multiple sexual partner’s relationships behaviors create a network that drives HIV transmission and the majority of STIs in the general population [39]. The low use of condom is among the factors for the high prevalence of unprotected sexual intercourse among young adults and more especially adolescents [40]. Discussion about sexual health is sensitive, and an embarrassing for adolescents because they are still learning and negotiating to adapt and maintain the intimate relationship at their very first time in life [41, 42]. Also, teaching sexual health topics to adolescents may be incompatible with cultural norms, especially for adolescent girls who are not confident to disclose their sexual life ambitions, feelings, and preferences [42, 43]. Nevertheless, the immaturity of the prefrontal cortex in adolescents is associated with heightened impulsivity and inability to have a wise plan or forecast the consequences of risk-taking behaviors [44]. In some studies, more than 50% of adolescents had never discussed with their sexual partner about safer sex, including condom use [45–47]. About two-thirds of HIV/AIDS-related infections and mortality in the world occur in SSA [12], and adolescents and young adults comprise more than one-third of its total population (49). Therefore, the high prevalence of sexual intercourse and especially sex with multiple partners among adolescents may continue to drive HIV/AIDS pandemic in this region if we don’t take quick measures regarding the adolescent’s sexual health policy.

Furthermore, our current study has provided evidence of some similarities between the five countries regarding the prevalence of sexual intercourse behaviors among adolescents. Except for Tanzania, there were relatively small differences in the study findings across the countries because of differences in the socio-economic structure, cultural, political, legal, and religious domains. For instance, teaching or discussions about sexual and reproductive health may be incompatible with cultural norms in some countries especially for adolescent girls who have limited social interaction and less confidence to disclose their sexual ambitions [41, 48].

Moreover, the current study revealed that adolescents who engaged in sexual intercourse were more likely to have other risk-taking behaviors such as smoking cigarettes, alcohol use, and illicit drug use (marijuana and amphetamines). Adolescents are curious in exploring the unknown and venturing a number of risk-taking behaviors [42] including sexual intercourse and substance use. Therefore, the strong evidence of correlations between sexual intercourse and other risk-taking behaviors in our current study suggest the clustering of health risk-taking behaviors among adolescents. For instance, the adolescent who drinks alcohol or uses illicit drugs may have a sensation-seeking of other risk-behavior such as sexual intercourse. The correlation between the clustering of risk-taking behaviors such as substance use has been shown to drive the sexual intercourse behaviors among adolescents in SSA [27, 43].

We also found that female adolescents who had parental connectedness were significantly less likely to have had sexual intercourse or sex with multiple partners. Family communication and parental connectedness provide protection against risk-taking behaviors among adolescents (52). For instance, studies in SSA have reported that adolescents living with neither parent had a lower likelihood of delaying sexual intercourse [44]. Moreover, other studies have reported that adolescent from families with poor parental connectedness has increased the likelihood of involving in risk-taking behaviors [45–47]. The comparable findings from our current study suggest an independent role of parental connectedness as a social protective factor against sexual intercourse behaviors among adolescents and more especially female adolescents.

The current study has the following strength; although it was a cross-sectional study, we used appreciably large sample size pooled from five countries. Moreover, the finding gives valuable insight and scope of the association between sexual and non-sexual risk-taking behaviors in the region with the highest burden of HIV/AIDS. Therefore, our study informs the policymakers to oversee the policy and programs that target multiple domains regarding clustering of risk-taking behaviors among adolescents.

On the other hand, the current study should be interpreted with a consideration of the following possible limitations. First, the GSHS study involves in-school adolescents, therefore, the current finding, may not be generalized to include out-of-school adolescents. Out-of-school adolescents may have relevant information regarding risk-taking behaviors. Therefore, current study findings cannot be generalized to all adolescents. Second, since the design of the study was cross-sectional, hence, this limits its utility to explain the relative proportion and temporal correlates of all explanatory factors of the sexual intercourse behaviors among adolescents. Third, since the survey was self-reported, it relied upon study participants recall memory hence leading to potential recall bias. Fourth, most of the study variables are based on a single item-response. Therefore, it could have unfairly reflected a complex concept behind the clustering of risk behaviors among adolescents. Fifth, social desirability could be another limitation of the current study. Most questions addressed undesirable and sensitive behavior like sexual intercourse. Adolescents could have responded less of sensitive questions, while responded more of questions that target good behaviours, hence, leading to response bias. Therefore, there could be a possibility of under-reporting sensitive or bad behaviors while over-reporting good behaviors. Although we used the principle of anonymity in the current study, still the issue of social desirability is indisputable.

## Conclusions

Sexual intercourse and more especially sex with multiple partners were prevalent and strongly correlated with substance use among in-school adolescents in SSA. However, the correlation was higher among female adolescents than male adolescents. A customized public health intervention that targets multiple risk factors concurrently may benefit adolescents with clustering of sexual and non-sexual risk-taking behaviors. Furthermore, parental connectedness may be an effective social promoting factor for sexual health among female adolescents.

## Supplementary information

**Additional file 1: Table S1.** Summary of sampling technique, data collection, and sample size of the Global School-based Students Health Survey of the five Sub Saharan Africa countries.

## Data Availability

The datasets files used for analysis in the current study are publicly free available in the WHO repository (https://www.who.int/ncds/surveillance/gshs/datasets/en/).

## Abbreviations

AIDS: Acquired Immune Deficiency Syndrome
aOR: Adjusted odds ratio
CDC: Centre for Diseases Control and Prevention (USA)
COR: Crude odds ratio
GSHS: Global School-based Student Health Survey
HIV: Human Immunodeficiency Virus
SSA: Sub-Saharan Africa
UNAIDS: United Nations Programme on HIV/AIDS
UNESCO: United Nations Educational, Scientific and Cultural Organization
UNICEF: United Nations Children Fund
USA: United States of America
WHO: World Health Organization
