# Supplementary material for: Prevalence and correlates of sexual intercourse among sexually active in-school adolescents: an analysis of five sub-Sahara African countries for the adolescent’s sexual health policy implications
Source: BMC Public Health. 2019 Oct 12;19:1285. doi: 10.1186/s12889-019-7632-1 (PMC6790023; doi:10.1186/s12889-019-7632-1)
Supplement: Supplementary file 1 — Additional file 1: Table S1. Summary of sampling technique, data collection, and sample size of the Global School-based Students Health Survey of the five Sub Saharan Africa countries. [file 12889_2019_7632_MOESM1_ESM.docx]

Additional file 1: Table 1 Summary of sampling technique, data collection, and sample size of the Global School-based Students Health Survey of the five Sub Saharan Africa countries

| Country | Year | Sample description | | Overall response rate (%) | Sample size |
| --- | --- | --- | --- | --- | --- |
|  |  | School-level | Class level |  |  |
| Benin | 2016 | Schools contain grades 6 to 1 were included in the sampling frame. Schools were selected systematically with the probability proportional to enrolment in grades 6 to 1 using a random start. | Classes with the majority of students in grades 6 to 12 were included in the sampling frame. Systematic equal probability sampling with a random start was used to select classes from each school that participates in the survey. | 78 | 2536 |
| Mozambique | 2015 | Schools contain grades 8 to 12 were included in the sampling frame. Schools were selected systematically with the probability proportional to enrolment in grades 8 to 12 using a random start. | Classes with the majority of students in grades 8 to 12 were included in the sampling frame. Systematic equal probability sampling with a random start was used to select classes from each school that participates in the survey. | 80 | 1918 |
| Namibia | 2013 | Schools contain grades 7 to 12 were included in the sampling frame. Schools were selected systematically with the probability proportional to enrolment in grades 7 to 12 using a random start. | Classes with the majority of students in grades 7 to 12 were included in the sampling frame. Systematic equal probability sampling with a random start was used to select classes from each school that participates in the survey. | 89 | 4531 |
| Seychelles | 2015 | Schools contain grades S1 to S5 were included in the sampling frame. Schools were selected systematically with the probability proportional to enrolment in grades S1 to S5 using a random start. | Classes with the majority of students in grades S1 to S5 were included in the sampling frame. Systematic equal probability sampling with a random start was used to select classes from each school that participates in the survey. | 82 | 2540 |
| Tanzania | 2014 | Schools contain grades 6 to 7, and Form 1 to 3 was included in the sampling frame. Schools were selected systematically with the probability proportional to enrolment in grades 6 to 7, and Form 1 to 3 using a random start. | Classes with the majority of students in grades 6 to 7 and Form 1 to 3 were included in the sampling frame. Systematic equal probability sampling with a random start was used to select classes from each school that participates in the survey. | 87 | 3793 |
| Total sample size | | | | | 15,318 |
